# Controller design and experimental validation of walking for a musculoskeletal bipedal lower limb robot based on the spring-loaded inverted pendulum model

**DOI:** 10.3389/frobt.2024.1449721

**Published:** 2024-11-05

**Authors:** Yiqi Li, Yelin Jiang, Koh Hosoda

**Affiliations:** ^1^ Graduate School of Engineering Science, Osaka University, Osaka, Japan; ^2^ Graduate School of Engineering, Kyoto University, Kyoto, Japan

**Keywords:** McKibben-type pneumatic artificial muscle (PAM), musculoskeletal biped robot, spring-loaded inverted pendulum model, PAM model identification, model-based PAM driven controller, robot walking experiment, ground reaction force

## Abstract

In the study of PAM (McKibben-type pneumatic artificial muscle)-driven bipedal robots, it is essential to investigate whether the intrinsic properties of the PAM contribute to achieving stable robot motion. Furthermore, it is crucial to determine if this contribution can be achieved through the interaction between the robot’s mechanical structure and the PAM. In previous research, a PAM-driven bipedal musculoskeletal robot was designed based on the principles of the spring-loaded inverted pendulum (SLIP) model. The robot features low leg inertia and concentrated mass near the hip joint. However, it is important to note that for this robot, only the design principles were based on the SLIP model, and no specialized controller was specifically designed based on the model. To address this issue, based on the characteristics of the developed robot, a PAM controller designed also based on the SLIP model is developed in this study. This model-based controller regulates ankle flexion PAM to adjust the direction of the ground reaction force during robot walking motion. The results indicate that the proposed controller effectively directs the leg ground reaction force towards the center of mass during walking.

## 1 Introduction

In robotics research, the McKibben pneumatic artificial muscle (PAM) is a soft actuator that has garnered significant attention. This type of actuator is distinguished by its excellent mechanical compliance and numerous advantageous characteristics. It is a lightweight, direct-drive actuator that boasts a very high force-to-weight ratio compared to other types of actuators, such as electric motors and hydraulic actuators Tondu (2012), Kalita et al. (2022). Additionally, it is highly backdrivable, allowing for flexible adaptation to external forces.

Due to these properties, PAMs have been deployed in the development of various bipedal robots, enabling these robots to demonstrate diverse dynamic performances. Hosoda et al. demonstrated how antagonistic pneumatic actuators in a musculoskeletal lower-limb bipedal robot can facilitate three types of human-like dynamic locomotion by employing a trial-and-error method Hosoda et al. (2008). Similarly, Niiyama et al. developed a musculoskeletal bipedal lower-limb robot named Athlete Robot, which is driven by PAMs. This robot is capable of achieving an impressive running motion, controlled by human muscle activation patterns derived from the muscle activity and kinetic data of human movements Niiyama et al. (2012). These robots are designed to mimic the human skeletal structure based on the principles of bionics.

As muscle-like actuators, PAMs are frequently used in the design of bipedal robots to mimic human functions and understand human biomechanics. A. Rosendo and X. Liu have developed a bipedal robot utilizing PAMs and provided roboticists with biomimetic concepts for controlling robot locomotion, as discussed in Rosendo et al. (2015) and Liu et al. (2018), contributing to the enhancement of the human reflex system.

Remarkably, these robots are designed to mimic the human skeletal structure based on the principles of bionics. They have achieved various dynamic motions, such as walking and jumping, using simple control strategies based on rules of thumb or less complicated control systems that rely on straightforward modeling.

Due to the inherent delayed and nonlinear characteristics of the PAM, controlling a PAM-driven robot cannot be achieved using complex model control methods like those used for motor-driven robots, such as Model Predictive Control. To advance research on the control of bipedal lower-limb robots driven by PAMs, it is essential to investigate whether the inherent characteristics of PAMs can contribute to the realization of stable robot movements. Additionally, it is crucial to determine whether such contributions can be achieved through the interaction between the robot’s mechanical structure and the PAMs.

In this research, a musculoskeletal bipedal lower-limb robot driven by PAMs was proposed to utilize the mechanical properties of the spring-loaded inverted pendulum (SLIP) model. The SLIP model, initially proposed by Blickhan (1989), is notable for being one of the most fundamental yet adaptable template models Seipel and Holmes (2007). Its applicability extends to both walking and running dynamics Geyer et al. (2006), Pelit et al. (2020), Iqbal et al. (2021). Despite their simplicity, these models offer a robust framework for analyzing bipedal locomotion in both humans and animals Müller et al. (2016), Burns et al. (2023).

There have been numerous studies on the control of multi-joint and humanoid robots using the SLIP model. X. Xiong et al. introduced a method for generating dynamic walking gaits with the SLIP model and applied it to a simulated humanoid in their work Xiong and Ames (2020). G. Garofalo et al. and J. Chang et al. have utilized the dynamics of the bipedal SLIP model as a template for controlling a fully actuated, multi-joint, 5-link robot through simulation studies in their respective works Garofalo et al. (2012), Chang et al. (2021). These studies exclusively validated the SLIP model-based controllers in simulations and did not involve their implementation on real robots. Furthermore, no robots were designed or developed based on the SLIP model in these studies.

Unlike traditional motor-driven bipedal robots, the lightweight and direct-drive attributes of PAMs enable the minimization of leg mass, allowing for a greater concentration of mass in desired areas.

In previous research, we developed a bipedal robot driven by PAMs that fully utilizes their characteristics. This robot features legs with very low inertia and concentrates 83% of the robot’s mass near the hip joint Li et al. (2024). However, due to the zigzag shape of the robot’s legs, a specialized controller must be designed to manage the PAMs. Therefore, based on the above characteristics of the developed robot, this study proposes a controller based on the SLIP model to regulate the direction of the ground force during the walking motion. This ensures that the robot’s ground reaction force directed toward its center of mass (COM), which is close to the hip joint, following the SLIP model.

The rest of this paper is organized as follows: Section 2 presents the detailed controller design, while the musculoskeletal bipedal robot system platform is introduced in Section 3. Section 4 outlines the PAM model used in this study and describes the PAM identification experiment. The details of the robot walking experiments and the experimental results are provided in Sections 5, 6, respectively. Finally, Section 7 provides a comprehensive discussion based on the results.

## 2 Controller design based on the SLIP model

This section outlines the design of the controller for pneumatic artificial muscles (PAM) to regulate the direction of the ground reaction force (GRF) based on the SLIP model. The controller design disregards the mass and rotational inertia of the legs, treating the robot as a center of mass and at the hip joint, consistent with the SLIP model.

### 2.1 Dynamics of the proposed musculoskeletal bipedal robot

**FIGURE 1:**
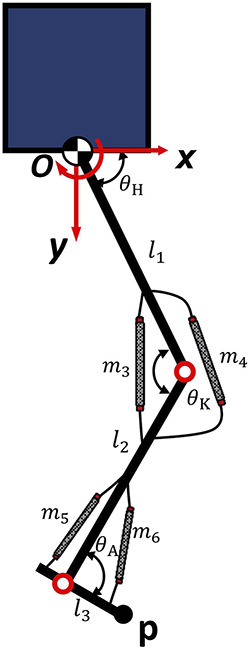
Leg kinematics of the Robot.

Figure 1 illustrates the schematic construction of the proposed robot leg and its kinematics. The coordinate system is displayed in the figure, with the origin located at the hip joint. The clockwise (CW) direction of rotation is defined as the positive direction. Considering the endpoint of the bipedal robot’s foot as $p={\left[p_{x},p_{y}\right]}^{\text{T}}$ , calculated by Equation 1 and given that the robot operates within a two-dimensional plane with its center of mass primarily located at the hip joint, this study focuses on the knee and ankle joints. Here, the hip joint angle, denoted as $\theta_{H}$ , is a known value. The angular vector for these joints is denoted as $\theta ={\left[\theta_{K},\theta_{A}\right]}^{\text{T}}$ .

$$\begin{array}{cc} & p_{x}=l_{1}\cos\theta_{H}-l_{2}\cos\left(\theta_{K}-\theta_{H}\right)+l_{3}\cos\left(\theta_{A}-\theta_{K}+\theta_{H}\right)\\ & p_{y}=l_{1}\sin\theta_{H}+l_{2}\sin\left(\theta_{K}-\theta_{H}\right)+l_{3}\sin\left(\theta_{A}-\theta_{K}+\theta_{H}\right) \end{array}$$ (1)

$\Delta p={\left[\Delta p_{x},\Delta p_{y}\right]}^{\text{T}}$ and $\Delta \theta ={\left[\Delta \theta_{K},\Delta \theta_{A}\right]}^{\text{T}}$ represent small displacements in the foot endpoint p and joint angles θ , respectively. The relationship between these two displacements is defined using the Jacobian matrix J , as follows:

Δp=JΔθ (2)

The relationship between the joint torque $\tau ={\left[\tau_{K},\tau_{A}\right]}^{\text{T}}$ and the reaction force from joint torques $f={\left[f_{x},f_{y}\right]}^{\text{T}}$ can be derived using the force Jacobian matrix, as follows:

$$\tau =J^{\text{T}}f$$ (3)

Given that the joint torque τ is generated by joint stiffness, it can be calculated using the joint stiffness matrix K and the joint angular displacement Δθ :

τ=KΔθ (4)

$$\begin{array}{c} K=diag\left(k_{K},k_{A}\right)=\left[\begin{array}{cc} k_{K} & 0\\ 0 & k_{A} \end{array}\right] \end{array}$$ (5)

Here, $k_{K}$ and $k_{A}$ in Equation 5 represent the stiffness coefficients of the knee and ankle joints, respectively. The relationship between the reaction force f and the displacement of the foot endpoint Δp can be derived from Equations 2, 3, 4 as follows:

$$f={\left(J^{\text{T}}\right)}^{-1}KJ^{-1}\Delta p$$ (6)

The matrix ${\left(J^{\text{T}}\right)}^{-1}KJ^{-1}$ , derived using the Jacobian matrix J and the joint stiffness matrix K , is a component of the equation presented in (Equation 6). This matrix serves as a transformed stiffness matrix used to calculate the linkage force from the spring. Equation 6 establishes the relationship between the force and the displacement of the joint angles.

In order to design based on the SLIP model, the relationship between the joint stiffness matrix K and the PAM force $F_{m}$ needs to be derived.

This study focuses on two primary joints: the knee and ankle joints. Each joint is actuated by two antagonistic PAMs. The moment arm matrix, denoted as $A_{m}$ , represents the geometric configuration of the PAM attachments around each joint, which influences the translation of PAM forces into joint torques. The mathematical expression of the relationship between PAM forces and the resulting joint torques is as follows:

$$\tau =A_{m}F_{m}$$ (7)

$$A_{m}=\left[\begin{array}{cccc} a_{m3} & -a_{m4} & 0 & 0\\ 0 & 0 & a_{m5} & -a_{m6} \end{array}\right]$$ (8)

Here, $a_{m3}$ and $a_{m5}$ , as well as $a_{m4}$ and $a_{m6}$ in Equation 8 are the moment arms of the monoarticular PAMs that exert positive and negative moments, respectively, on the joint. The PAM force vector is represented by $F_{m}={\left[Fm_{3},F_{m_{4}},F_{m_{5}},F_{m_{6}}\right]}^{\text{T}}$ , derived from the PAM model. This model will be further discussed in Section 4.

**FIGURE 2:**
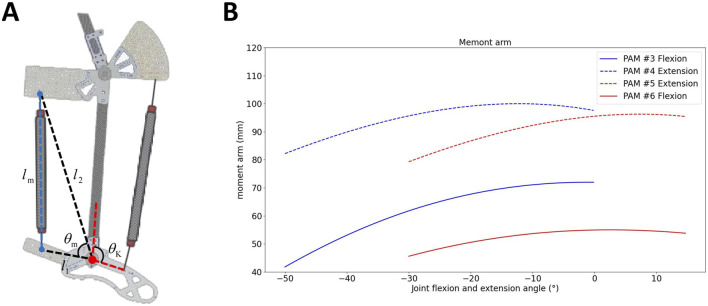
**(A)** Schematic for calculating the moment arm of a monoarticular. **(B)** Moment arm of flexion and extension PAMs in the knee and ankle joints.

The skeletal monoarticular PAMs span a joint, attaching to two links. The schematic for the moment arm mechanism analysis of one PAM is depicted in Figure 2A. The moment arm of one PAM, denoted as $a_{m}$ , can be derived using Equation 9, where $l_{1}$ , $l_{2}$ , and $\theta_{m}$ are parameters of the mechanical structure of an individual monoarticular PAMin the leg, and $l_{m}$ represents the length of the PAM.

$$\begin{array}{ll} a_{m} & =\frac{l_{1}l_{2}}{l_{m}}\sin\left(\theta_{m}\right)\\ l_{m} & =\sqrt{l_{1}^{2}+l_{2}^{2}-2l_{1}l_{2}\cos\left(\theta_{m}\right)} \end{array}$$ (9)

Figure 2B illustrates the moment arms of the flexion and extension PAMs in the knee and ankle joints at various angles. The figure demonstrates that the moment arm of the extensor PAM is greater than that of the flexion PAM in each joint.

The joint stiffness can be calculated as by using the moment arm matrix $A_{m}$ and the PAM force vector $F_{m}$ according to Equation 10.

$$K=\frac{\partial \tau }{\partial \theta }=\frac{\partial A_{m}F_{m}}{\partial \theta }$$ (10)

### 2.2 SLIP model based PAM controller design

This study focuses on the control of Pneumatic Artificial Muscles (PAMs) in each leg to generate forces aligned with the dynamics described by the SLIP model. The SLIP model is characterized by its ability to simulate the spring-like properties of legged locomotion, where the primary dynamics involve a spring compressing and decompressing to propel the body forward, similar to a pogo stick. The essential condition for achieving SLIP-like behavior is the following constraint:

$$p\times \left({\left(J^{\text{T}}\right)}^{-1}KJ^{-1}p\right)=0$$ (11)

In Equation 11, the variables consist of the joint angles vector $\theta ={\left[\theta_{K},\theta_{A}\right]}^{\text{T}}$ , which cannot be directly controlled, and the PAM air pressure vector $P_{m}={\left[P_{m3},P_{m4},P_{m5},P_{m6}\right]}^{\text{T}}$ that can be controlled directly. Given that there is only one Equation 11, the joint angles and air pressures of the three PAMs, obtained from real-time sensors, can be used to calculate the required air pressure for the remaining one PAM to fulfill this constraint.

In this study, the ankle joint, being the joint closest to the ground, plays a crucial role during leg support action. We choose to control the air pressure of the ankle flexion PAM #6 to satisfy the constraint by calculating the desired air pressure of PAM #6 using $\theta_{K}$ , $\theta_{A}$ , $P_{m3}$ , $P_{m4}$ , and $P_{m5}$ as known values.

## 3 Bipedal pneumatic musculoskeletal lower-limb robot

### 3.1 Robot design

**FIGURE 3:**
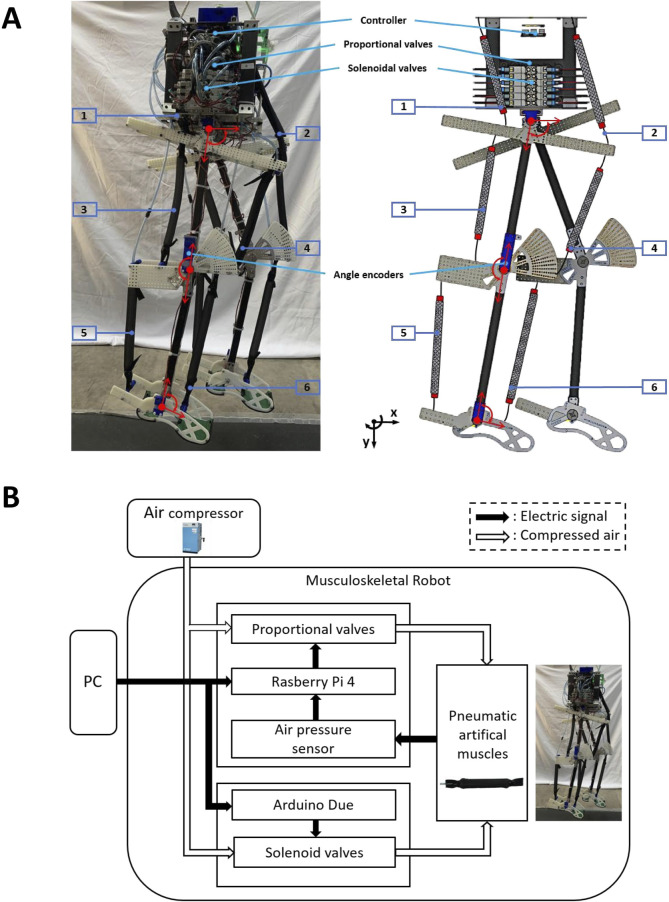
**(A)** Bipedal pneumatic musculoskeletal lower-limb robot developed based on the SLIP model. **(B)** Robot control system.

To validate the controller mentioned in Section 2, this study utilized a bipedal pneumatic musculoskeletal robot developed based on the SLIP model, as depicted in Figure 3A. This robot served as the platform for the robotic system. The robot is equipped with three hinge joints in each leg: the hip, knee, and ankle joints, restricting its movement to the sagittal plane. Each leg is independently actuated by six monoarticular, self-made PAMs.

The silicone tube portion of each PAM has a maximum contraction rate of about 30 percent, which primarily determines the joint mobility ranges as follows: the hip has a range of 10° in flexion and 10° in extension; the knee allows for 50° in flexion; and the ankle provides 30° in plantarflexion and 15° in dorsiflexion.

The detailed parameters and the function of each PAM used in the robot, as well as a comparison of the joint range of motion between the proposed robot and humans (referencing human lower limb joint range of motion gait analysis research Dicharry (2010) and Webster and Darter (2019)), can be found in the Supplementary Material.

The entire robot weighs 12 kg and stands at a height of 970 mm, mimicking the size of a small human. Since the PAM as an actuator is extremely lightweight and directly driven, the valves used for actuation can be centrally mounted on the body near the hip joint. Therefore, a significant portion of the robot’s weight (10 kg) is concentrated near the hip joint within a small-size compact body. This configuration results in very low leg inertia, aligning with the principles of mass-less legs and a point mass in the SLIP model. Such a design endows the robot with characteristics similar to those of the SLIP model, enabling the dynamics of the COM to effectively represent the overall dynamics of the entire robot. Consequently, only the dynamics of the COM need to be considered when designing the control strategy for this robot, simplifying the design of the controller.

### 3.2 Robot control system

Figure 3B shows the control system of the robot. The air pressure from the air compressor is 0.6 MPa. Each PAM is controlled by a proportional valve and a three-port solenoid valve. Proportional valves and air pressure sensors are utilized in a PID controller to regulate the internal air pressure of the PAMs, while the solenoid valves are used to provide rapid and dynamic performance. The control of both types of valves and the reception of data from the sensors are managed by a microcontroller and a control board.

The entire system reads sensor values and writes actuator commands at a rate of 200 Hz, except for the proportional valve, which is controlled at a frequency of 60 Hz due to hardware constraints. For more details about the robot’s PAM parameters and the control system, please refer to Li et al. (2024).

## 4 PAM modeling and identification experiment

### 4.1 Modeling of PAM

Various researchers have modeled Pneumatic Artificial Muscles (PAMs) from different perspectives Kelasidi et al. (2011), including geometrical Chou and Hannaford (1996) and empirical approaches Wickramatunge and Leephakpreeda (2013). Mohseni et al. developed a dynamic PAM model and demonstrated its accuracy in predicting the forces generated by PAMs Mohseni et al. (2020). In this study, we modeled our self-made PAM based on this established model, which is demonstrated in  Equations 12, 13.

$$F_{PAM}\left(P_{m},l_{m}\right)=P_{m}Ff_{la}\left(l_{m}\right)+Ff_{lp}\left(l_{m}\right)$$ (12)

where P represents the instantaneous air pressure, and $l_{m}$ denotes the length of the PAM. The parameter F is a constant representing the fixed gain associated with the force exerted by the PAM.

$$\begin{array}{cc} & f_{la}\left(l_{m}\right)=1+a_{0}l_{m}+a_{1}l_{m}^{2}\\ & f_{lp}\left(l_{m}\right)=c_{0}+c_{1}l_{m} \end{array}$$ (13)

where $a_{0}$ , $a_{1}$ , $a_{2}$ and $c_{0}$ , $c_{1}$ are parameters to be determined through experimental methods.

### 4.2 PAM identification experiment

**FIGURE 4:**
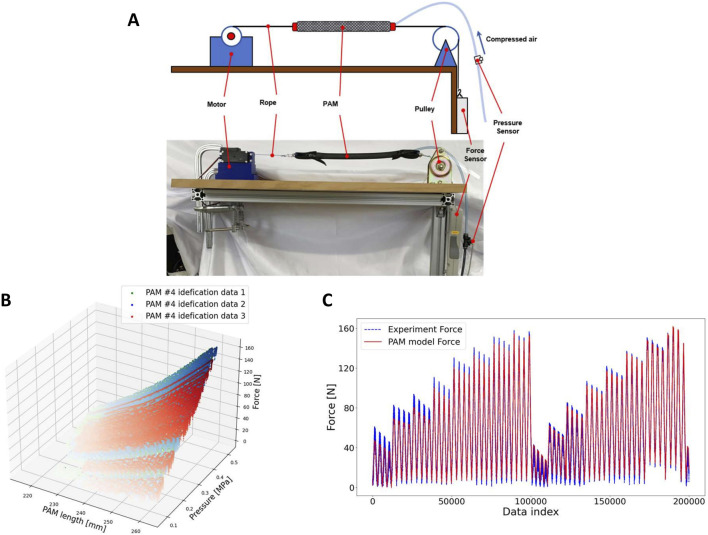
**(A)** Setup of PAM identification experiment. **(B)** PAM identification experimental data. **(C)** Experimental and fitting data.

The experimental setup used to determine the PAM parameters in this study is illustrated in Figure 4A. From left to right, the components include a servo motor (ds5160, ANNIMOS Servo Technology, China), a handmade PAM, and a force sensor (ZTA-500N, IMADA, Japan), connected using non-stretchable Dyneema rope. The air pressure within the PAM was measured by an air pressure sensor (PSE 540, SMC, Japan), and the length of the PAM was calculated based on the angle measured by an encoder and the radius of the motor’s rotation. A Raspberry Pi 4 was employed to control the setup and collect data at a frequency of 200 Hz.

In the PAM identification experiment, the rope was maintained in a slack state, and the PAM was inflated to an initial pressure ranging from 0.1 to 0.55 MPa. Subsequently, a force controller was used to drive the motor, stretching the rope to approximately 8 N. The rest length of the PAM was calculated using the angle encoder. The motor extended the PAM to various lengths at different, randomly determined rates; this process was repeated 20 times.

Figures 4B, C illustrate an example of experimental data from PAM identification experiments and the results fitted by the PAM model. The R-squared values for the model fitting results of the experimental data for each PAM exceeded 0.97.

### 4.3 Experimental testing of the PAM identified model

Experiments were conducted to evaluate whether the identified PAM models are compatible with the real-world applications of the developed robot. From Equation 7, the joint torque τ can be derived from the moment arm matrix $A_{m}$ and the PAM force vector $F_{m}$ . The moment arm matrix $A_{m}\left(\theta \right)$ varies as a function of the joint angle θ . Meanwhile, the PAM force vector $F_{m}\left(l_{m},P_{m}\right)$ is determined by the air pressure vector $P_{m}$ and the PAM length vector $l_{m}$ , as shown in Equation 14:

$$F_{m}\left(l_{m},P_{m}\right)={\left[F_{m_{3}},F_{m_{4}},F_{m_{5}},F_{m_{6}}\right]}^{\text{T}}$$ (14)

From Equation 9, since PAM length is a function of the joint angle, the PAM force vector can be redefined as a function of the joint angle θ and the air pressure vector $P_{m}$ , expressed as $F_{m}\left(\theta ,P_{m}\right)$ . Consequently, the joint torque τ can be rewritten as a function of both θ and $P_{m}$ , expressed as $\tau \left(\theta ,P_{m}\right)$ .

The antagonistic PAMs of the knee joint and ankle joint were inflated individually to a specified air pressure while the robot was suspended (not in contact with the ground). Considering the light mass of the robot’s legs and neglecting the inertia of the legs, the joint torque satisfies the following condition: $\tau \left(\theta ,P_{m}\right)={\left[\tau_{K},\tau_{A}\right]}^{\text{T}}=0$ . Therefore, given the air pressure in the antagonist PAMs of the joint, the joint angle can be determined.

In the experiment, the knee and ankle flexion PAM #3 and #6 were initially inflated to air pressures of 0.15, 0.35, and 0.55 MPa, respectively. Subsequently, the extensor PAMs #4 and #5 were gradually inflated from 0.1 to 0.6 MPa, with each inflation phase increasing the air pressure by 0.05 MPa, at intervals of 0.1 s. During this process, the angle data for the knee and ankle joints were recorded separately and compared with the joint angles calculated by the theoretical model mentioned above. Consequently, this process can be considered a quasi-static process.

**FIGURE 5:**
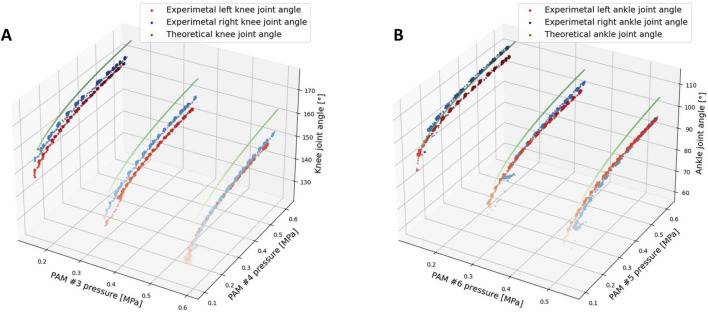
Joint angle result from experimental and theoretical results, RMSE values between theoretical and experimental results: Knee joint angle (left: 9.293, right: 7.597) and Ankle joint angle (left: 6.790, right: 6.750). **(A)** Knee joint angle comparison from experiment and theoretical model. **(B)** Ankle joint angle comparison from experiment and theoretical model.

Figure 5 illustrates the comparison of joint angles between the knee and ankle derived from the theoretical model and those obtained from experimental results. The root-mean-square deviation (RMSE) was used to quantify the difference between the theoretical and experimental results. The RMSE values are as follows: Knee joint angle (left: 8.493, right: 7.597) and Ankle joint angle (left: 6.790, right: 6.750).

From Figure 5 and the RMSE results, the discrepancy in the error magnitude between the left and right legs can primarily be attributed to errors in muscle production and mechanical assembly. For the knee and ankle comparisons, it is evident that the difference between the experimental and theoretical results for the knee angle is slightly larger compared to the ankle angle. The discrepancy observed may stem from the fact that the mass of the leg and foot for each limb was not accounted for in the theoretical model calculations, despite its relatively small weight. Nonetheless, this mass still influences the experimental results. In the theoretical analysis, joint angles were computed under the assumption of an equilibrium state, where the joint torque produced by the two PAMs was equal, thereby neglecting the mass of the shank and foot. However, during the experiments, the joint torque at the knee and ankle joints must also influenced by the weight of the shank and foot parts of the leg. For the ankle joint, only the mass of the foot contributes to the joint torque, whereas the knee joint is impacted by the torque generated by both the mass of the foot and the shank. Consequently, the knee joint is more significantly affected by the mass of the leg in comparison to the ankle joint, resulting in a greater divergence between the experimental and theoretical outcomes for the knee joint than for the ankle joint. Overall, the experimental and theoretical results exhibit a certain degree of similarity.

## 5 Musculoskeletal bipedal robot walking experiment

To validate the effectiveness of the proposed PAM control based on the SLIP model, a robot walking experiment was designed and implemented. The primary objective of this experiment was to evaluate the controller’s performance.

In this experiment, a force plate (Tec Gihan Co., Ltd., TF-3040) was used to measure the ground reaction force (GRF) during the walking trials.

### 5.1 Experiment setup

**FIGURE 6:**
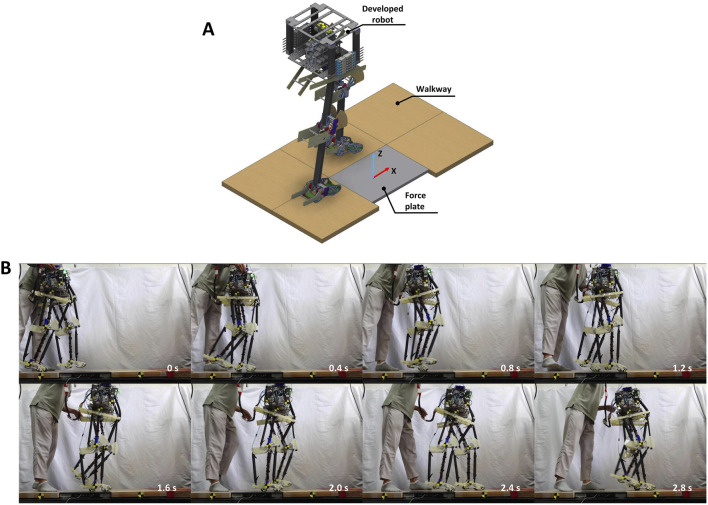
**(A)** Setup of the robot walking experiment. **(B)** Snapshot of the walking trial.

The experimental environment and procedure are illustrated in Figure 6A. Initially, the robot was held by the experimenter with its left leg positioned in front, not touching the ground, and its right leg behind, touching the ground. At this stage, the robot’s body was kept upright. The experiment began when the experimenter released the robot, allowing it to start walking forward. Upon the second contact of the right leg with the ground, it stepped on the force plate, and the GRF was measured and recorded. It is important to note that no additional constraining devices were used to restrict the robot’s movement exclusively to the sagittal plane during the experiment.

### 5.2 Walking drive pattern

**FIGURE 7:**
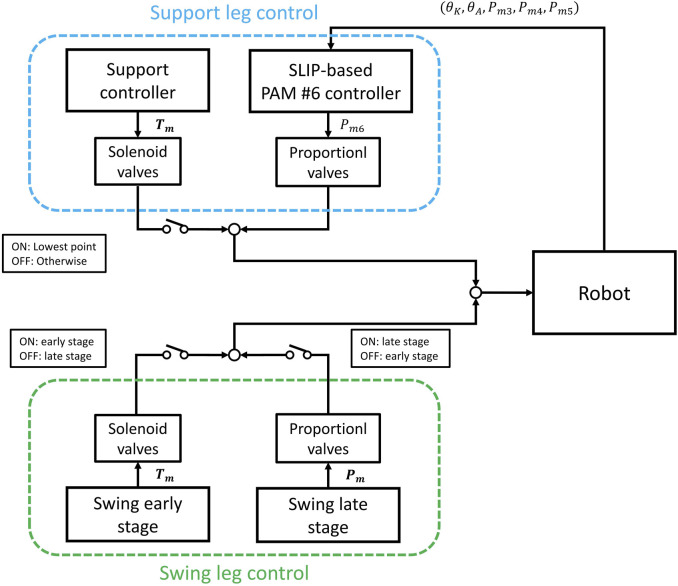
Robot walking control pattern.

The robot uses three control patterns for walking in this experiment: PAMs walking initialization, leg support control, and leg swing control. A switch-type touch sensor is integrated into the robot’s feet to identify leg touchdown and liftoff, enabling the determination of whether it is in the support control mode or the leg swing control mode. Figure 7 illustrates the control logic of the robot’s leg support and leg swing control.

#### 5.2.1 PAMs walking initialization

A PID controller was utilized to regulate the proportional valves, ensuring a specific initial air pressure for each PAM. The initialization process lasted 1 s, with the maximum permissible air pressure error limited to 5%. The initial air pressures for each PAM in the left and right legs are detailed in Table 1, with the left leg positioned forward and the right leg positioned backward in preparation for the walking motion.

**TABLE 1:** Initial air pressure of the PAMs.

Left PAM no.	Initial pressure (Mpa)	Right PAM no.	Initial pressure (Mpa)
#1	0.10	#1	0.50
#2	0.50	#2	0.10
#3	0.20	#3	0.20
#4	0.20	#4	0.15
#5	0.15	#5	0.30
#6	0.30	#6	0.30

#### 5.2.2 Support leg control

The support controller, executed by the solenoid valves shown in Table 2. Air was supplied to the knee and ankle joint extension PAMs, specifically PAMs #4 and #5, causing them to contract and generate a specific force that enhances the upward movement of the robot. The hip antagonist PAM, PAM #1, was supplied, and PAM #2 was exhausted to facilitate the forward movement of the robot.

**TABLE 2:** Leg support control.

PAM no.	Support action	Action time $\left(T_{m}\right)$
#1	Supply	80 ms
#2	Exhaust	80 ms
#3	Close	\
#4	Supply	50 ms
#5	Supply	50 ms
#6	Close	\

In addition, the ankle flexion PAM #6 is controlled by pressure PID using proportional valves, with the target pressure calculated by the controller designed based on the SLIP model in Section 2.

Upon detecting a touchdown, the sum of the ankle and knee joint angle values is then used to determine the moment when the robot reaches its lowest point. The lowest point is defined as the moment when the sum of these two joint angles reaches its minimum value.

Once the leg contacts the ground, the SLIP-based PAM #6 controller activates to regulate PAM #6 and manage the ground reaction force. Subsequently, at the moment when the touchdown leg reaches its lowest point, support control is activated, enabling the robot to move forward.

#### 5.2.3 Swing leg control

After the leg lifts off the ground, the leg swing control is activated. The swing leg control is divided into an early stage and a late stage, as outlined in Table 3. In the early stage, solenoid valves were used to rapidly inflate Flexion PAM #3 and #6 while deflating Extension PAM #4 and #5, allowing the leg to tuck in. At the same time, hip PAMs #1 and #2 act swiftly to swing the leg forward, concluding the early stage.

**TABLE 3:** Leg swing control.

PAM no.	Swing action (early stage)	Action time $\left(T_{m}\right)$	PID control $\left(P_{m}\right)$ (late stage)
#1	Exhaust	80 ms	0.10 Mpa
#2	Supply	80 ms	0.50 Mpa
#3	Supply	50 ms	0.20 Mpa
#4	Exhaust	50 ms	0.20 Mpa
#5	Exhaust	50 ms	0.15 Mpa
#6	Supply	50 ms	0.30 Mpa

In the late stage, proportional valves are controlled by a PID controller to regulate the PAMs in the swing leg to achieve the specified air pressure within a 200 ms timeframe, preparing the swing leg for the subsequent touchdown.

The walking experiment of the robot was conducted using the described control pattern. The robot successfully managed to take three steps forward without falling, despite limitations in the size of the experimental site and cables.

Given the difficulty in ensuring the robot starts walking from the exact same initial position each time, six trials were conducted, and the average values were calculated to represent the results. The force plate had a sampling rate of 1 kHz, whereas the robot’s data sampling frequency was 200 Hz.

A camera (RX100VII, Sony Co.) was used to capture the robot’s sagittal plane movements through a transparent plate at 120 frames per second (fps). Snapshots of the walking experiment, captured at 0.4 s intervals, are depicted in Figure 6B.

## 6 Experimental result

### 6.1 Data on robot joint angles and PAM pressures during the walking experiment

**FIGURE 8:**
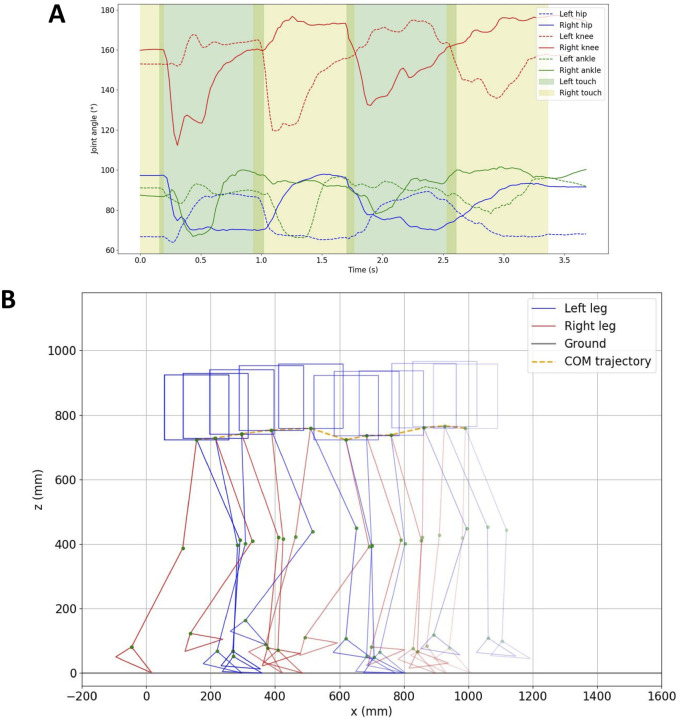
**(A)** Hip, knee, and ankle joint angles during the walking experiment. The green and yellow sections represent when the left and right legs touch the ground, respectively. **(B)** Trajectory of the robot’s walking path (derived from joint angles and COM trajectory, depicted every 360 ms).

Figure 8A illustrates the variation in the angles of the joints in the left and right legs during the walking experiment. The segments highlighted in green and yellow indicate when the left and right legs make contact with the ground, respectively. It can be seen that the joint angles of both legs change periodically with the walking cycle.

From the data, the walking gait cycle (the sum of the swing phase and stance phase) for a single leg is as follows: left leg: 1.571 s, right leg: 1.577 s. The swing phase duration is 0.712 s for the left leg and 0.729 s for the right leg. The stance phase duration is 0.859 s for the left leg and 0.848 s for the right leg. Each leg’s contact duration with the ground is approximately 850 ms, with one leg being supported for around 770 ms and both two legs being supported for about 80 ms.

The COM data was acquired by analyzing the robot’s motion using Kinovea Charmant (2021). The robot’s movement trajectory was determined by integrating the COM data with the robot’s joint angle information recorded from the installed angle encoders. In Figure 8B, the robot’s walking path is depicted. The COM of the robot travels a distance of approximately 0.835 m over a duration of around 3.2 s, achieving an average speed of roughly 0.26 m/s.

**FIGURE 9:**
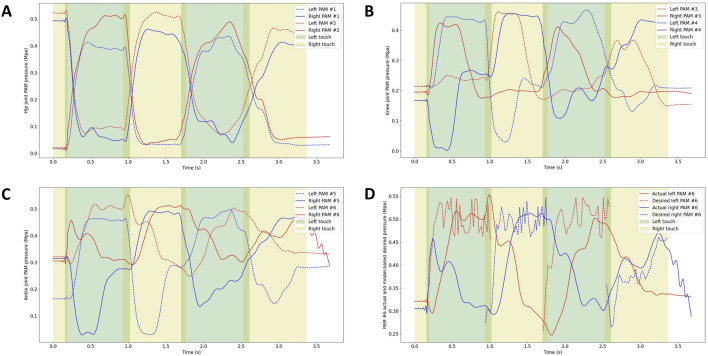
Air pressure of the antagonist PAMs in each joint of the left and right legs: **(A)** Air pressure of antagonist PAMs in the hip joint (Front swing: PAM #2, Back swing: PAM #1). **(B)** Air pressure of antagonist PAMs in the knee joint (Flexion: PAM #3, Extension: PAM #4). **(C)** Air pressure of antagonist PAMs in the ankle joint (Flexion: PAM #6, Extension: PAM #5). **(D)** Desired air pressure of PAM #6 calculated by the model and actual air pressure when the right and left legs touch the ground, respectively.

The air pressure fluctuations during walking are depicted in Figure 9. In Figures 9A–C, the antagonist PAM air pressures in each joint of both the left and right legs are shown, demonstrating systematic fluctuations in air pressure for each PAM corresponding to different walking states of the legs. Additionally, Figure 9D displays the desired air pressure for PAM #6, as determined by the model, and the actual air pressure measured during ground contact of the left and right legs. The desired pressure for PAM #6 was calculated using the control method derived from the SLIP model. The noticeable fluctuations in the desired air pressure of PAM #6 can be attributed to the nonlinearity present in the computational model.

Observing the results depicted in the figure, it is evident that the actual air pressure closely follows the model calculations, albeit with some delay. This delay is attributed to the time lag in the action of the proportional valve and the supply and exhaust processes of the PAM.

### 6.2 Data from the force plate

**FIGURE 10:**
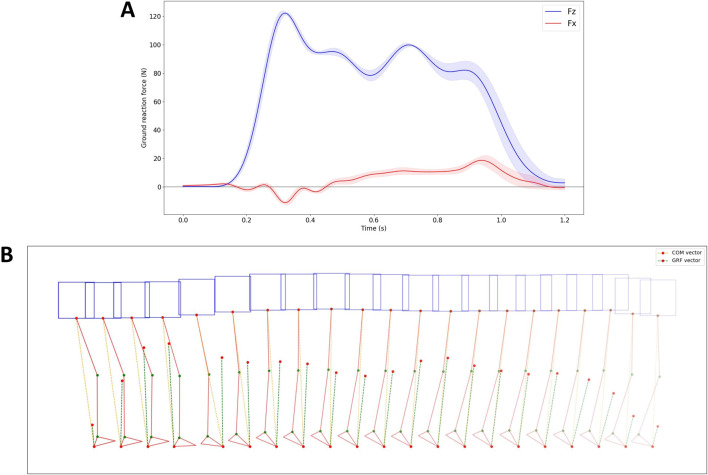
Ground reaction force data, along with data on COM and GRF vectors, while applying the proposed controller. **(A)** GRF data in the *X* and *Z* axis. **(B)** The robot’s pose, as well as the COM and GRF vectors during the second touchdown of the right leg, depicted every 40 ms.

A low-pass filtering method with a cutoff frequency of 50 Hz was implemented to minimize the influence of noise in the GRF data. The results for the *X*-axis and *Z*-axis GRFs are displayed in Figure 10A.

The vertical and horizontal shapes of the GRFs closely resemble the patterns observed in human walking and SLIP models as discussed in the study by Mauersberger et al. Mauersberger et al. (2022). The vertical GRFs (shown in blue), exhibit an M-shape for each stride, oscillating around the robot weight of 12 kg. On the other hand, the horizontal GRFs (depicted in red) are characterized by an initial negative phase, representing “braking” to decelerate the body mass, followed by a switch in polarity (circles) signifying forefoot “push-off” that accelerates the body mass forward and upward.

Figure 10B illustrates the robot’s pose, the COM vector, and the GRF vector during the second touchdown of the right leg. In the figure, the orange dotted line indicates the direction of the COM, extending from the point of ground contact of the foot to the COM point. The green dotted line represents the direction of the GRF, with the length of the vector reflecting its magnitude. The figure demonstrates that during the second right leg touchdown, the GRF direction closely aligns with the COM direction.

To verify the effectiveness of the proposed controller based on the SLIP model, an additional experiment was conducted. In this experiment, a walking trial was performed by the robot without using the model-based PAM #6 controller, which meant that during walking, the valves controlling PAM #6 were closed, and no inflation or deflation operations were performed on PAM #6 during the contact phase of the supporting leg with the ground. All other conditions remained unchanged. The data from this walking trial were collected and compared to the data from the trial using the proposed controller to assess the differences.

**FIGURE 11:**
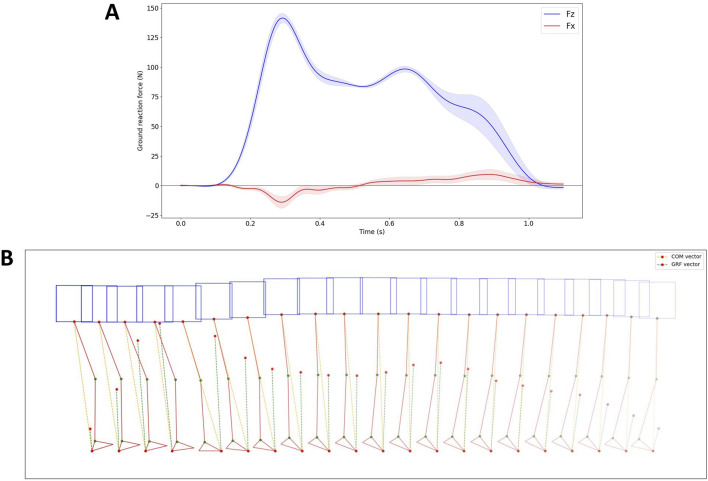
Ground reaction force data and COM and GRF vector data without the application of the proposed controller. **(A)** GRF data in the *X* and *Z* axis. **(B)** The robot’s posture, along with the COM and GRF vectors, during the second touchdown of the right leg, depicted every 40 ms.

The results are displayed in Figure 11. The GRF without the controller exhibits a similar shape but a smoother profile compared to when the controller is active. This smoother profile is due to the lack of active control of PAM #6 in both legs during leg touchdown. Interestingly, the difference between the direction of GRF and COM is more noticeable when the controller is not utilized compared to when it is active.

Without using the proposed controller, the robot walked with a single gait cycle of 1.456 s, with an average duration of 0.711 s for the swing phase and 0.745 s for the stance phase. The contact time of each leg with the ground was approximately 740 ms, with one leg being supported for approximately 700 ms and both two legs being supported for approximately 40 ms.

In order to visually assess the directional difference between the GRF vector and the COM vector under various control scenarios, the angle between these two vectors was calculated using Equation 15.

**FIGURE 12:**
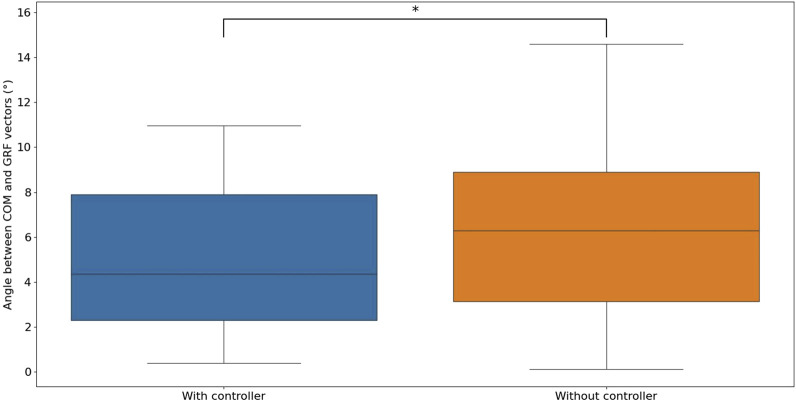
The statistics of the angle between the COM and GRF vectors with and without the proposed controller are as follows: With the proposed controller: 5.093 ± 3.327 degrees (°), without the proposed controller: 6.443 ± 4.274 degrees (°).

Statistical analysis was conducted on these angles with and without the proposed controller, as shown in Figure 12. The findings revealed a significant decrease in the angle between the GRF vector and the COM vector when the controller was activated compared to when it was not active. The mean, variance, and maximum values of the angle between the GRF and COM vectors are significantly smaller when the controller is used compared to when it is not used.

$$angle=∠\left(C\vec{O}M,G\vec{R}F\right)$$ (15)

## 7 Discussion

In previous studies, we developed and built a musculoskeletal bipedal robot using Pneumatic Artificial Muscles (PAMs) as actuators. By employing lightweight PAMs and a leg design that minimizes weight, we effectively reduced the inertia of the robot’s legs. This configuration concentrates the center of mass in a compact body around the hip joint, leading to lower leg inertia in accordance with the principles of the SLIP model Li et al. (2024). However, based on the above characteristics of the developed robot, no specialized PAM controller has been developed to align the zigzag legs of the robot with the SLIP model.

In the design of the controller, the mass and rotational inertia of the legs are ignored, and the robot is treated as a center of mass, the same as in the SLIP model. Due to the ankle joint being the joint closest to the ground, the antagonist PAMs of the ankle joint play a crucial role during leg support action. In this research, we have developed an ankle flexion PAM #6 controller inspired by the SLIP model to regulate the PAM and control the ground reaction force during the robot’s support leg motion while walking. This controller ensures that the robot’s GRF aligns with its COM, adhering to the essential principle outlined in the SLIP model.

To effectively control the robot using the proposed method, a PAM identification experiment was designed and conducted to determine the model of each PAM. Based on the identified PAM model, experimental testing was performed, as detailed in Section 4.3, to compare the joint angles calculated by the PAM model with the actual measured joint angles. This evaluation aimed to assess the compatibility of the identified PAM model with the practical application of the developed robot. The results show a certain degree of similarity between the experimental and theoretical outcomes.

And, the robot walking experiments were conducted using the proposed control method to evaluate its performance. From Figure 8, it can be seen that the average speed of the robot was 0.26 m/s. The average duration of a single walking gait cycle for both legs of the robot was 1.574 s, with 0.721 s for the swing phase and 0.853 s for the stance phase.

From the force plate results in Figures 10A, 11A, it can be observed that the GRFs exhibit patterns in both the vertical and horizontal directions that closely resemble those observed in human walking and SLIP model. A comparison of the ground reaction force data and the robot’s pose data for robot walking with and without a controller reveals that the angle between the GRF vector and the COM vector is significantly smaller when the controller is engaged compared to when it is disengaged. This demonstrates that the proposed PAM controller, based on the SLIP model, effectively regulates ground reaction force.

The durations of the swing phase without the proposed controller and with the proposed controller are almost the same (without proposed controller: 0.711 s, with proposed controller: 0.721 s), but the duration of the stance phase with the controller (0.853 s) is significantly longer than the 0.745 s in the stance phase without the proposed controller. The time for double legs support has also been reduced from about 80 ms with the controller to about 40 ms without the controller.

It can be seen that the proposed controller, which regulates the internal pressure of PAM 6 for the support leg, extends the single leg support duration and the double leg support period. Although it was possible to make the robot walk without applying the proposed controller in the experiment, the shorter support time of the single leg and double legs led to unstable walking of the robot.

However, in addition to the discrepancy between the real robot and the SLIP model led to some deviations. With the maximum angles between the GRF and COM vectors typically observed when the knee and ankle joint extension PAMs were active during the leg support phase. This is due to the PAM is slow to respond and cannot reach the desired air pressure value quickly enough. The deviation becomes smaller during the rest period of the leg support phase, after the knee and ankle extension PAMs have completed their active movement.

There are still some shortcomings and areas that need improvement. For simplicity in the controller design, it is assumed that the toes of the robot foot remain in contact with the ground during the leg support phase. However, in the actual experimental process, the toe may not always be in contact with the ground, which can lead to deviations. Due to the limitations of the PAM identification device’s performance, the modeling in this study did not consider the damping term influenced by the rate of change in the length of the PAM. This damping term can have an effect when the PAM changes rapidly, for example, when employing model-based control to regulate the PAM for executing jumping motion. Additionally, the PAM also exhibits a hysteresis phenomenon during inflation and deflation Abu Mohareb et al. (2021), which may affect the control accuracy of the PAM.

In this study, when designing the controller, only the direction, not the magnitude, of the ground reaction force was considered for control. In future research, new controllers can be designed to control an additional PAM, such as the extension PAM #3 of the knee joint, to regulate the magnitude of the ground reaction force.

By controlling the ground reaction force, it becomes feasible to achieve variable stiffness performance different from that of the spring legs in the SLIP model with fixed spring stiffness. This adjustment can lead to the robot exhibiting various dynamic characteristics during walking.

## Data Availability

The raw data supporting the conclusions of this article will be made available by the authors, without undue reservation.
