# Supplementary material for: Controller design and experimental validation of walking for a musculoskeletal bipedal lower limb robot based on the spring-loaded inverted pendulum model
Source: Front Robot AI. 2024 Nov 5;11:1449721. doi: 10.3389/frobt.2024.1449721 (PMC11574207; doi:10.3389/frobt.2024.1449721)
Supplement: Supplementary file 1 [file DataSheet1.pdf]

Supplementary Material

1 SUPPLEMENTARY TABLES AND FIGURES

1.1 Tables

Table S1. Arrangement of the PAMs

no.	PAM length (mm)	tube length (mm)	function
#1	310	260	hip extension
#2	360	310	hip flexion
#3	344	250	knee flexion
#4	350	300	knee extension
#5	380	280	ankle extension
#6	396	270	ankle flexion

Table S2. Comparison of Joint Range of Motion Between Robot and Human

Joint range of motion	Robot (flexion / extension)	Human (flexion / extension)
Hip joint pitch	+10 $\sim$ -10°	+20 $\sim$ -20°
Knee joint pitch	+50 $\sim$ 0°	+60 $\sim$ 0°
Ankle joint pitch	+30 $\sim$ -15°	+20 $\sim$ -5°
